# Long non-coding RNA NHEG1/hsa-miR-665/HMGB1 axis is involved in the regulation of neuroblastoma progression

**DOI:** 10.1080/21655979.2021.1983277

**Published:** 2021-12-10

**Authors:** Yuqing Zhang, Yuping Hu, Aihong Pan, Lei He, Jin Wang, Fangfang Zhou, Yongbo Lei, Yuanyuan Wang

**Affiliations:** a Department of Pediatrics,The first people’s Hospital of Hefei, South District Binhu Hospital of Hefei First People’s Hospital, Neonates Department, Hefei, China; b Nursing Department,The first people’s Hospital of Hefei, South District Binhu Hospital of Hefei First People’s Hospital, Nursing Department, Hefei, China; c Graduate school,Institute of Nursing, Far Eastern University, Master in Art of Nursing, Far Eastern University, Sampaloc, Manila, Philippines; d Department of Pediatrics, Maternal and Child Health Hospital of Weifang City, Maternal and Child Health Hospital of Weifang City, Weifang, China

**Keywords:** Lncrna NHEG1, miR-665, HMGB1, neuroblastoma

## Abstract

Long non-coding (lncRNA) neuroblastoma highly expressed 1 (NHEG1) has been reorganized as a prognostic factor in neuroblastoma (NB), but the molecular mechanisms in the suppression of neuroblastoma remain to be elucidated. In our study, we explored the functional roles of lncRNA NHEG1 in neuroblastoma and the underlying molecular mechanism. We collected NB tumor samples and adjacent normal tissues to compare lncRNA NHEG1 expression. Through bioinformatic target prediction, we selected potential downstream effectors of lncRNA NHEG1 for functional validation in NB cell lines. We observed that lncRNA NHEG1 was significantly upregulated in NB tissues as compared to the normal tissues. In NB tissues, lncRNA NHEG1 expression showed an inverse correlation with hsa-miR-665 (miR-655), but a positive correlation with high mobility group box 1 (HMGB1). In NB cell lines, lncRNA NHEG1 knockdown caused the upregulation of miR-665 and the downregulation of HMGB1. Through a series of functional assays, we further demonstrated that lncRNA Nheg1 knockdown suppressed cell proliferation, migration and invasion of NB cells, which could be rescued by miR-665 inhibitor and HMGB1 overexpression. Together, our data demonstrated that lncRNA NHEG1 serves as a competitive partner to negatively regulate the activity of miR-665, which relieves the inhibition on HMGB1 expression and promotes the aggressive phenotype of neuroblastoma cells. Our study indicates that lncRNA NHEG1/miR-665/HMGB1 axis may play an important role in regulating the aggressiveness and the progression of neuroblastoma.

## Introduction

Neuroblastoma (NB) is the most common extracranial solid tumor in children, which accounts for approximately 15% of pediatric cancer-related deaths [1]. Despite the intensive research into the molecular mechanisms controlling NB progression and the recent development of multimodal therapy [2–4], the prevalence of high-risk NB cases characterized by rapid progression, poor prognosis and high mortality rate poses a serious challenge in clinical treatment [1,5]. Therefore, understanding the molecular mechanism to explore the potential therapeutic targets and mechanisms of neuroblastoma.

Emerging evidences have demonstrated that long non-coding RNAs (lncRNAs) play essential roles in the development and progression of multiple types of cancers [6–8]. As an example, lncRNA neuroblastoma highly expressed 1 (NHEG1) was a lncRNA which is predicted to regulate a network of microRNAs in human lung adenocarcinoma [9]. In 2020, Zhao et al. [10] further demonstrated the oncogenic role of lncRNA NHEG1 in NB, which is associated with poor prognosis in NB patients. Mechanistically, lncRNA NHEG1 physically interacts with and stabilizes DEAD-box helicase 5 (DDX5) by preventing its degradation, which leads to β-catenin transactivation and promotes NB progression [10]. However, other potential downstream targets of lncRNA NHEG1 in NB and their mechanisms in regulating NB progression have not been fully explored.

MicroRNAs (miRNAs) are a class of small non-coding RNAs with 20–23 nucleotides in length [11,12]. miRNAs function to target the complementary or a partial complementary sequence in the 3ʹ untranslated regions (3ʹ UTR) of the target mRNA to negatively regulate its expression, leading to either mRNA degradation or translational inhibition [13–15]. miRNAs are important regulators in a multitude of biological processes, including cell proliferation, apoptosis, cell differentiation, cellular development, and the progression of cancers [13–16]. The first evidence of miRNA implication in cancer came from its deregulation in human Chronic Lymphocytic Leukemia [17]. Later accumulating evidence has demonstrated that miRNA dysregulation plays an important role in cancer progression [18–21]. For example, miR-15a-5p, miR-15b-5p, and miR-16-5p were reported to inhibit NB progression by directly targeting Neuroblastoma MYC Oncogene (MYCN) [20]. hsa-miR-665 (miR-655) has emerged as an important regulator different type of cancers [13,22,23]. It was reported as tumor repressor in neuroblastoma [13]. In ovarian cancer miRNA‑665 suppresses the growth and migration of cancer cells by negatively regulating Homeobox Protein Hox-A10 (HOXA10) [22]. miR-665 is also expressed in the exosomes derived from hepatocellular carcinoma, which was proposed as a marker for the diagnosis and prognosis [23]. Moreover, recent studies showed that miR-665 acts as a tumor-suppressive miRNA in retinoblastoma by directly targeting high mobility group box 1 (HMGB1) [24,25]. In NB, HMGB1 is highly expressed and considered as a tumor-promoting factor [26]. However, whether miR-665/HMGB1 axis regulates the progression of NB remains to be elucidated.

In this study, we first collected NB tumor samples and adjacent normal tissues to compare lncRNA NHEG1 expression. We observed the upregulation of lncRNA NHEG1 in NB tissue as well as NB cell line. Through bioinformatic prediction, miR-665 was found as a potential target of lncRNA NHEG1, and HMGB1 was a downstream target of miR-665. We therefore hypothesized that miR-665/HMGB1 axis may play an important role to mediates the effect of lncRNA NHEG1 in NB cells. We showed that lncRNA NHEG1 regulated the expression of miR-665 and HMGB1. Through different functional assays in NB cell lines, we further demonstrated that lncRNA NHEG1 knockdown suppressed cell proliferation, migration and invasion, which was rescued by miR-665 inhibitor and HMGB1 overexpression. Our study suggests that lncRNA NHEG1/miR-665/HMGB1 axis may play an important role in regulating the aggressiveness and progression of neuroblastoma.

## Materials and methods

### Patient recruitment and sample collection

In this study, a total number of 60 patients diagnosed with NB (high-risk subtype, 36 males and 24 females, 8 months to 5 years 2 months, 2.6 ± 0.8 years) were enrolled. All the patient tumor samples and adjacent normal tissues were collected by surgery between July 2015 and April 2018 in South District Binhu Hospital of Hefei first people’s Hospital. The diagnosis of NB was confirmed by histopathological examinations. Inclusion criteria: patients were with the initial NB diagnosis without therapy, and the usage of children’s biospecimens and related clinical data were approved by their parents. Exclusion criteria: patients were diagnosed with congenital malformations or other clinical disorders. This study and the usage of patient samples were approved by the Ethics Committee of South District Binhu Hospital of Hefei first people’s Hospital, Hefei, China (170,918–03). All the parents of 60 neuroblastoma patients signed the written informed consent. The clinicopathological information of all patients is summarized in Table 1.

**Table 1.** The specific primer for the qRT-PCR

Primer		Sequences (5ʹ-3)
LncRNA NHEG1	F R	GATTTCAGCGCGTTCATTGC CAGCCTCACATCTGCATTCC
MiR-665 U6	F R F R	GCCGAGACCAGGAGGCUGA CTCAACTGGTGTCGTGGA GCTTCGGCAGCACATATACTAAAAT CGCTTCACGAATTTGCGTGTCAT
HMGB1	F R	TGCAGATGACAAGCAGCCTT GCTGCATCAGGCTTTCCTTT
GAPDH	F R	GGAGCGAGATCCCTCCAAAAT GGCTGTTGTCATACTTCTCATGG

### Cell culture

Human neuroblastoma cell line SK-N-BE, SH-SY5Y, SK-N-SH, LAN-6 and normal dorsal root ganglia cells (DG) were used in this study. All the cells were purchased from the Cell Bank of Chinese Academy of Sciences (Shanghai, China). Cells were cultured in 1:1 mixture of Ham’s F12 medium (10% FBS and 2 mM Glutamine, Thermo Fisher Scientific, 31,765,035) and Dulbecco’s Modified Eagle’s Medium (DMEM high glucose, pyruvate, no glutamine, Thermo Fisher Scientific, 21,969,035). Cells were incubated at 37°C in a humidified atmosphere of 5% CO_2_.

### Quantitative reverse transcription PCR (qRT-PCR)

RNA from all tissues and cells were extracted using Trizol reagent (15,596,026, Thermo Fisher Scientific) according to the manufacturer’s instructions. Purified total RNA was dissolved in DEPC water and the concentration was measured by NanoDrop spectrometer. Five microgram of total RNA was used for reverse-transcription into cDNA by RevertAid First Strand cDNA Synthesis Kit (K1622, Thermo Fisher Scientific). The resulted cDNA was quantified in the StepOnePlus™ Real-Time PCR System (Applied Biosystems, Carlsbad, CA, USA) using SYBR premix EX TAQ II kit (RR820A, Takara, Dalian, China). The cycling condition for qPCR: 95 ^o^C 2 mins, 40 cycles of 95°C 30 sec, 60°C 30 s and 72°C 60 s, with signal detection at the end of each cycle. For relative gene expression analysis, 2–∆∆Ct method was used to quantify the relative expression level and GAPDH was used as the internal reference gene. All primer sequences were synthesized and purchased from Shanghai Sangon Biotechnology Co., Ltd. (Shanghai, China), which are listed in Table 1.

### Transient cell transfection

Small interfering RNAs (si-lncRNA NHEG1#1, si-lncRNA NHEG1#2 and negative control si-NC), microRNA targeting oligonucleotides (MiR-665-mimic, miR-665-inhibitor, and negative control miR-NC), and pcDNA3.1-HGMB1 expression vector were synthesized by Shanghai Sangon Biotechnology Co., Ltd. (Shanghai, China) (See Table 2 for all the sequences). Cell lines SK-N-BE and SH-SY5Y were used for transfections. Cell transfection was performed using Lipofectamine® 3000 reagent (Thermo Fisher Scientific, L3000001). Briefly, cells were seeded in 6-well plates at a density of 5×10^5 cells/well. Twenty-four hours later, 100 nm of each molecule was added into 100 µL Opti-MEM® I Reduced-Serum Medium (Invitrogen, 31,985,062), and then 6 µL Lipofectamine 3000 reagent was added into the solution for 10 min incubation. The mixture was added to the cell culture dropwise and transfected cells were cultured for 48 h before subsequent experiments.

**Table 2.** The sequences of lncRNA NHEG1 siRNA, miR-665 mimic, miR-665 inhibitor, and HGMB1 siRNA

Primer	Sequences (5ʹ-3)
Si-lncRNA NHEG1#1 Si-lncRNA NHEG1#2 Si-NC	GCAUGUUGUUGACUGAUAACA GGAGAUAUUUCCUGAUAGACU CCCAGUUACGAATCGCUUCCA
miR-665 mimic miR-665 inhibitor miR-NC	ACCAGGAGGCUGAGGCCCCU AGGGGCCUCAGCCUCCUGGU UCGCUUGGUGCAGGUCGGGAA

### CCK-8 cell proliferation assay

Cell Counting Kit-8 (Dojindo Molecular Technologies, CK04) was used for cell proliferation assay. Forty-eight hours after transfection, cells were seeded in to a 96-well plate at a density of 3000 cell/well and cultured in a humidified cell culture incubator for 0, 24, 48, 72 and 96 h, respectively. Subsequently, 10 μL CCK8 reaction solution was added to the cell culture at indicated time point and incubated for 1 h in a humidified cell culture incubator. The light absorption value (OD value) in each condition was captured at 450 nm wavelength on a Synergy H1 microplate reader (Winooski, Vermont, USA).

### Colony formation assay

SK-N-BE, SH-SY5Y cells were seeded into a 6-well plate (1000 cells/well) and cultured for 14 days. The culture medium was changed every 3 days during the period. After 14 days, cells were fixed with 4% paraformaldehyde at room temperature for 10 mins and stained with 0.5% crystal violet (Sigma, 109,218) for 45 mins. After wash with PBS, the number of colonies was counted and photographed under Leica AM6000 microscope.

### Cell migration and invasion assays

For cell migration and invasion assay, the 24-well cell culture plates with 8-μm micropore inserts were used. Transwell upper chamber (Corning, #3401) without Matrigel (BD Biosciences, 356,234) was used for migration assay, while transwell upper chamber coated with the 50 μL Matrigel (1:5 dilution in PBS) was used for invasion assay. A total number of 1 × 10^5^ cells in cell migration assay or 2 × 10^5^ cells in cell invasion assay were inoculated into the upper chamber in serum-free medium and 500 μL of 10% serum-containing medium was added to the lower chamber. After 18 hours, cells were fixed with 4% paraformaldehyde at room temperature for 10 mins and stained with 0.5% crystal violet (Sigma, 109,218) for 45 mins. The number of migrating or invading cells in the transwell inserts was counted under Leica AM6000 microscope.

### Target prediction using bioinformatic tools

To search for the potential targets of lncRNA NHEG1, the sequence of lncRNA NHEG1 was retrieved from in NCBI gene database (https://www.ncbi.nlm.nih.gov/gene). The NHEG1 sequence was subject to miRNA prediction using LncBase Predicted v.2 Module of DIANA Tools [27] (http://carolina.imis.athena-innovation.gr/diana_tools/web/). The following criteria were used: ‘search by location’ and enter ‘chr6:136,982,165–136,993,234’, select ‘human’ – – enter ‘NHEG1’), and the prediction was performed ‘based on transcripts’. The threshold was set to ‘0.7’. The results can be found in the link below: http://carolina.imis.athena-innovation.gr/diana_tools/web/index.php?r=lncbasev2%2Findex-predicted&loc_search=1&location=chr6%3A136982165-136993234&location_search_type=0&threshold=0.8&filters=0&page=2

To identify hsa-miR-665, TargetScan 7.2 database (http://www.targetscan.org/vert_72/) [28] was used and the search result can be found in the link below:

http://www.targetscan.org/cgi-bin/targetscan/vert_72/targetscan.cgi?species=Human&gid=&mir_sc=&mir_c=&mir_nc=&mir_vnc=&mirg=mir-665

### Dual luciferase activity assay

To demonstrate the functional interaction between lncRNA NHEG1 or HMGB1 with miR-665, the sequences containing the wild-type binding site of miR-665 or the sequence with mutated binding site at lncRNA NHEG1 3ʹUTR or HMGB1 3ʹ UTR were cloned into the PmirGLO firefly luciferase respectively (Promega, E1330). Reporter Firefly luciferase plasmid and Renilla luciferase (hRlucneo) control plasmid were co-transfected with either miR-665 mimic or miR-NC in a 12-well plate (1 × 10^5 cells/well, SK-N-BE and SH-SY5Y cells) using Lipofectamine 3000 reagent (Invitrogen, L3000001). Forty-eight hours after transfection, relative Firefly and Renilla luciferase activities were measured by Dual-Luciferase Reporter Assay Kit (Promega, E1910) on a luminescence microplate reader (Infinite 200 PRO; Tecan). The relative Firefly luciferase activity in the reporter plasmid was normalized to that of Renilla luciferase (hRlucneo) control plasmid.

### Western blot

Cells were collected 48 hours after transfections and total protein were extracted with RIPA Lysis Buffer (Beyotime Biotechnology, P0013B). Protein concentration was quantified by a BCA Protein assay kit (Beyotime Biotechnology, P0009). 10 µg total protein was used for SDS-PAGE electrophoresis. Proteins in SDS-PAGE gel were then transferred onto a PVDF membrane (BioRad, 1,620,177). The membrane was blocked with 5% skimmed milk for 1 h and incubated with primary antibodies: HMGB1 (Abcam, ab18256, 1:1000 dilution) and GAPDH (Abcam, ab9485, 1:1500 dilution) overnight at 4°C. The membrane was washed 3 times with TBST buffer and further incubated with HRP-conjugated secondary antibody (Cell signaling #7074, 1:3000 dilution) at room temperature for 1 h. Then the membrane was washed 4 times with TBST and the protein bands were visualized using an enhanced chemiluminescence kit (Santa Cruz, sc-2048). The densitometry analysis was performed with Image J software.

## Statistical analysis

Three independent experiments were performed for each assay. The statistical difference between two groups was compared using unpaired student’s t tests. Comparisons among multiple groups were analyzed using one-way analysis of variance (ANOVA) with Tukey’s post hoc test for pairwise comparison. Comparisons of data at multiple time points were examined using two-way ANOVA. Spearman correlation analysis was performed to determine the correlation between the expression levels of two genes. Kaplan Meier Curve and log-rank test were used to compare the overall survival (OS) and progression-free survival (PFS) between lncRNA NHEG1 high expression and low-expression NB patients. Data were presented as mean ± standard deviation (SD). *P* < 0.05 was considered to be statistically different. GraphPad Prism 7.0 software was used for statistical analyses.

## Results

In our study, we explored the functional roles of lncRNA NHEG1 in NB and the underlying molecular mechanism. We observed that lncRNA NHEG1 was significantly upregulated in NB tumor and cell lines. Silencing lncRNA NHEG1 attenuated the aggressive phenotype of NB cells. We further revealed that miR-665/HMGB1 axis was involved in the regulation of the malignant phenotype of NB cells, which mediates the downstream effect of lncRNA NHEG1. Collectively, our study suggests that lncRNA NHEG1/miR-665/HMGB1 axis is involved in regulating the aggressiveness NB cells, which may be implicated in the progression of NB.

## LncRNA NHEG1 is upregulated in NB

**Figure 1.**
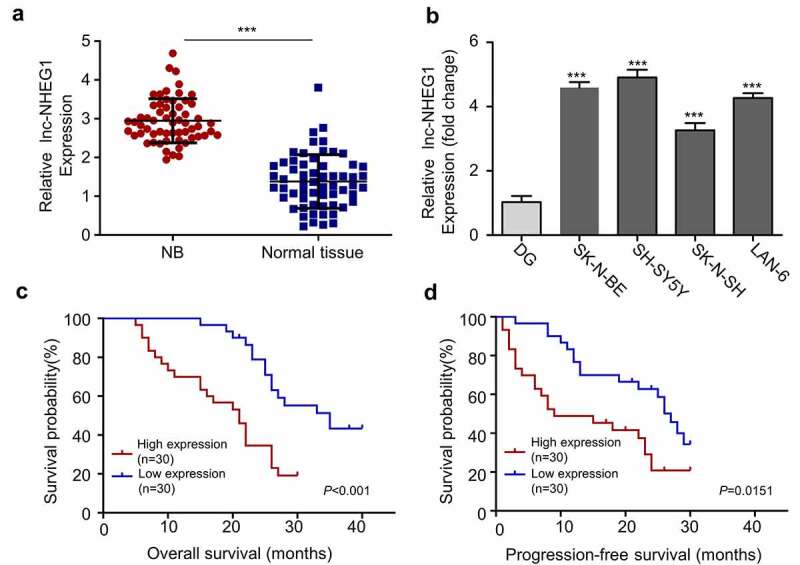
LncRNA NHEG1 expression is significantly upregulated in NB tissues and cell lines. A. LncRNA NHEG1 expression level in NB tumor samples and adjacent normal tissues (n = 60), *** *P* < 0.001. B. LncRNA NHEG1 expression level in NB cell lines (SK-N-BE, SH-SY5Y, SK-N-SH, LAN-6) and normal dorsal root ganglia cell (DG). (n = 3 experiments). *** *P* < 0.001 vs. DG cells. C-D. Kaplan Meier curve and log-rank test were used to compare the overall survival (OS) and progression-free survival (PFS) between lncRNA NHEG1 high expression (n = 30) and low expression NB patients (n = 30)

In order to investigate the expression level of lncRNA NHEG1 in NB, tumor samples and adjacent normal tissues were collected from 60 NB patients. qRT-PCR was performed to compare the relative lncRNA NHEG1 expression. Our results showed that lncRNA NHEG1 was significantly upregulated in NB tumors tissues as compared to the adjacent non-cancer tissues (Figure 1a). Likewise, we also observed the upregulation of lncRNA NHEG1 in human NB cell lines (SK-N-BE, SH-SY5Y, SK-N-SH, LAN-6) as compared to normal dorsal root ganglia cells (DG) (Figure 1b), with a relatively higher expression level detected in SK-N-BE and SH-SY5Y cells. Moreover, the median expression level of lncRNA NHEG1 was used as the cutoff to divide the NB patients into high expression (n = 30) and low expression (n = 30) group. Kaplan Meier curve analysis revealed that high expression of lncRNA NHEG1 was significantly correlated with a worse overall survival (OS) and progression-free survival (PFS) (Figures 1c, d). High lncRNA NHEG1 expression was also associated with a higher frequency of metastasis (Table 3). Together, this data indicates a potential role of lncRNA NHEG1 upregulation in NB progression. SK-N-BE and SH-SY5Y NB cell lines with high lncRNA NHEG1 expression were selected for subsequent functional studies.

**Table 3.** Summary of clinicopathological information of all patients included in this study

Factors		lnc-NHEG1 expression		P value
		Low (n = 30)	High (n = 30)	
Age				0.176
	≥1 year	22	17	
	<1 year	8	13	
Gender				0.6048
	Male	17	15	
	Female	13	15	
INSS Stage				0.0449
	I+ II+IVS	5	12	
	III+IV	25	18	
Risk group				0.0285
	Low+Intermediate	6	14	
	High	24	16	
Shimada Classification				0.2466
	uFH	21	15	
	FH	8	12	
	Missing	1	3	
Marrow Status				0.0384
	Metastasis	20	12	
	NM	10	18	

INSS: International Neuroblastoma Staging System; uFH: Unfavorable Histology; FH: Favorable Histology; NA: Not Amplified; NM: Not Metastasis.

## Silencing lncRNA NHEG1 inhibits NB cell proliferation, migration, and invasion

**Figure 2.**
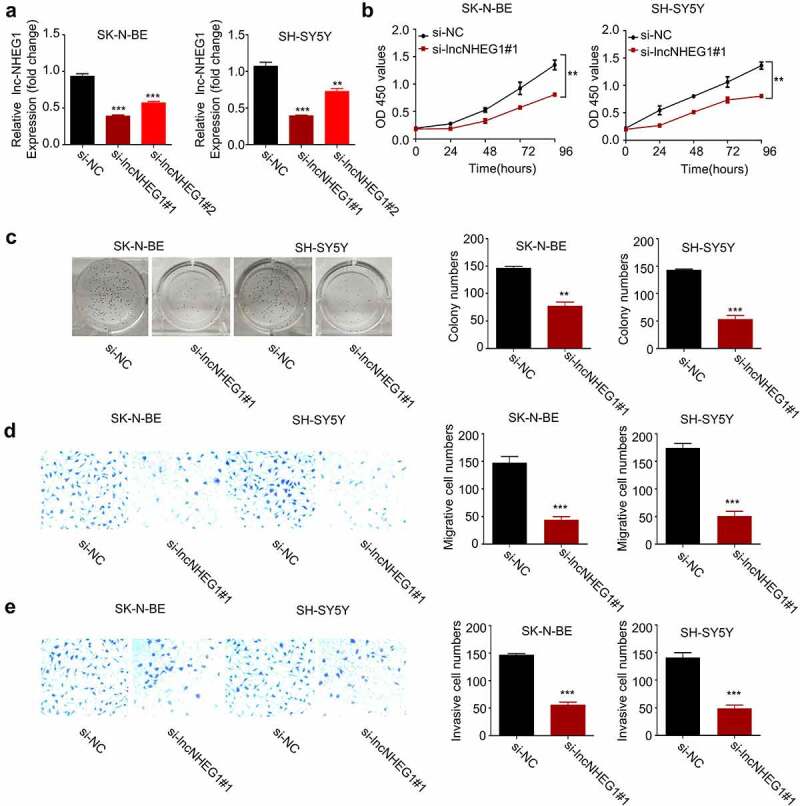
Silencing lncRNA NHEG1 inhibits proliferation, migration and invasion in NB cells. A. qRT-PCR analysis of lncRNA NHEG1 expression level in NB cells (SK-N-BE, SH-SY5Y) after transfection with si-lncNHEG1 #1 and #2. B. CCK-8 cell proliferation assay of NB cells treated with si-lncNHEG1#1. C. Colony formation assay in NB cells treated with si-lncNHEG1#1. D. Cell migration assay in NB cells treated with si-lncNHEG1#1. E. Cell invasion assay in NB cells treated with si-lncNHEG1#1. All experiments were performed 3 times and data were presented as mean ± SD. **P < 0.01 vs. si-NC group. ***P < 0.001 vs. si-NC group

To validate the functional role of lncRNA NHEG1 in supporting malignant phenotype of NB cells, we applied small interfering RNAs (siRNAs) to knock down lncRNA NHEG1 in SK-N-BE and SH-SY5Y cell lines. Both si-lncRNA NHEG1 #1 and #2 showed efficient knockdown effect on lncRNA NHEG1, and si-lncRNA NHEG1 #1 with a stronger silencing effect was selected for the following experiment (Figure 2a). We next performed CCK-8 cell proliferation assay and colony formation assay after silencing lncRNA NHEG1. We found that downregulation of lncRNA NHEG1 significantly inhibited cell proliferation (Figure 2b) and impaired the clonogenic capacity of NB cells (Figure 2c). We further analyzed the migration and invasion ability of NB cells after silencing lncRNA NHEG1 by transwell experiments. Our results demonstrated that lncRNA NHEG1 knockdown suppressed the migration and invasion of NB cells (Figures 2d, e). These data collectively indicate that the high expression level of lncRNA NHEG1 is indispensable for supporting the aggressive phenotype of NB cells.

## LncRNA NHEG1 targets miR-665 and negatively affects its expression

**Figure 3.**
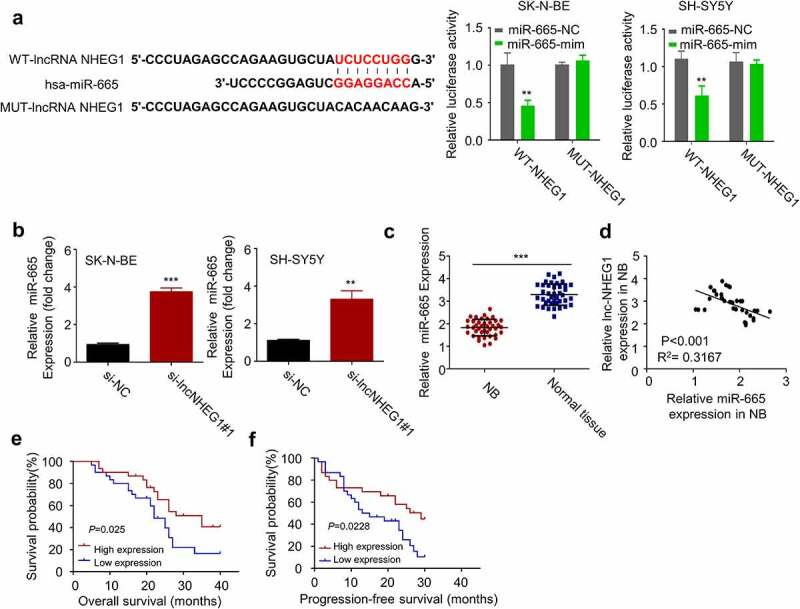
miR-665 was a downstream target of lncRNA NHEG1. A. Predicted binding site of miR-665 at the 3ʹUTR of lncRNA NHEG1. Wildtype binding site (WT-NHEG1) or mutated sequence (MUT-NHEG1) was cloned into luciferase reporter and dual luciferase reporter assay was performed in the presence or absence of miR-665 mimic (mim). n = 3 experiments. **P < 0.01 vs. miR-665-NC. B. miR-665 expression level was detected by qRT-PCR after transfection with si-lncNHEG1#1 in NB cells. n = 3 experiments, **P < 0.01 vs. si-NC group, ***P < 0.001 vs. si-NC group. C. miR-665 expression levels in NB tissues and adjacent normal tissues (n = 60). ***P < 0.001 vs. normal tissue. D. The correlation between lncRNA NHEG1 expression and miR-665 expression among the 60 NB tumor samples. E-F. Kaplan Meier curve and log-rank test were used to compare the overall survival (OS) (e) and progression-free survival (PFS) (f) between miR-665 high expression (n = 30) and miR-665 low expression NB patients (n = 30)

To identify the potential downstream miRNAs of lncRNA NHEG1, we relied on the DINAN tools (see methods for details). Among the predicted targets, hsa-miR-665 (miR-655) binding site was found at the 3ʹ untranslated region (UTR) of lncRNA NHEG1 (Figure 3a). In addition, recent studies showed that miR-665 acts as a tumor-suppressive miRNA in retinoblastoma by HMGB1 [24,25]. Therefore, we selected miR-665 as a candidate target of lncRNA NHEG1 for further investigation. We performed dual-luciferase reporter assay using the construct containing wildtype miR-665 binding sequence of lncRNA NHEG1 (WT-NHEG1) as well as the mutated sequence (MUT-NHEG1). In both SK-N-BE and SH-SY5Y cells, the transfection of miR-665 mimic significantly suppressed the luciferase activity of WT-NHEG1, which was not observed in the MUT-NHEG1 (Figure 3a). These results indicate the functional interaction between miR-665 and lncRNA NHEG1. We further demonstrated that miR-665 expression level was significantly upregulated after lncRNA NHEG1 knockdown in SK-N-BE and SH-SY5Y cells (Figure 3b). The expression level of miR-665 was also lower in NB tissues as compared to that of the adjacent non-cancer tissues (Figure 3c). Pearson correlation analysis further revealed a significant negative correlation between the expression levels of miR-665 and lncRNA NHEG1 in the NB tumor samples (Figure 3d). Kaplan Meier curve analysis revealed that high expression of miR-665 was correlated with a better overall survival (OS) and progression-free survival (PFS) in NB patients (Figures 3e, f). Together, these data suggest that lncRNA NHEG1 targets miR-665 and negatively regulates its expression.

## HMGB1 is a downstream target of miR-665

**Figure 4.**
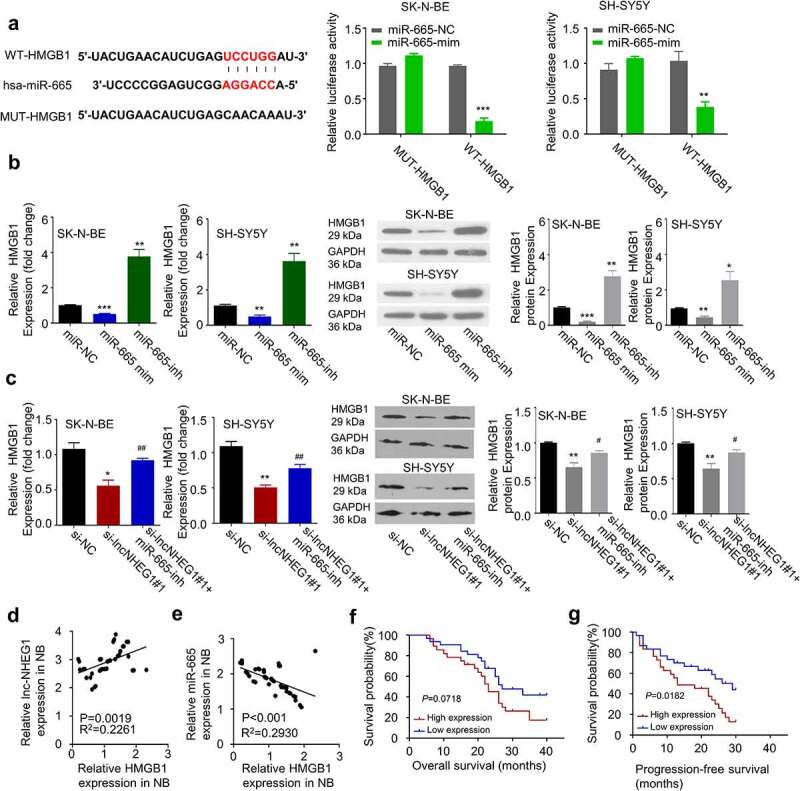
LncRNA NHEG1 knockdown decreased HMGB1 expression via sponging miR-665. A. Predicted miR-665 binding site in HMGB1 3ʹUTR. Wildtype binding site (WT-HMGB1) or mutated sequence (MUT-HMGB1) was cloned into luciferase reporter and dual luciferase reporter assay was performed in the presence or absence of miR-665 mimic (mim). n = 3 experiments. **P < 0.01 or ***P < 0.001 vs. miR-665-NC. B. HMGB1 expression level was determined by qRT-PCR and western blot after the treatment with miR-665 mimic or miR-665-inhibitor in NB cells. n = 3 experiments. *P < 0.05, **P < 0.01, ***P < 0.001 vs. mir-NC group. C. HMGB1 expression levels was determined by qRT-PCR and western blot after the treatment with si-lncNHEG1#1 or in the combination with miR-665-inhibitor. n = 3 experiments. *P < 0.05 vs. si-NC group, **P < 0.01 vs. si-NC group, #*P* < 0.05 vs. si-lncNHEG1#1 group, ##P < 0.01 vs. si-lncNHEG1#1 group. D. The correlation between lncRNA NHEG1 expression and HMGB1 expression in 60 NB tumor samples. E. The correlation between miR-665 expression and HMGB1 expression in in 60 NB tumor samples. F-G. Kaplan Meier curve and log-rank test were used to compare the overall survival (OS) (f) and progression-free survival (PFS) (g) between HMGB1 high expression (n = 30) and HMGB1 low expression NB patients (n = 30)

We next sought to find the potential downstream target of miR-665. We searched for miR-665 targets using TargetScan 7.2 database (see methods for details), and found 3ʹ 3ʹ UTR of HMGB1 mRNA contained a potential binding site for miR-665 (Figure 4a). Previous studies have also implicated HMGB1 as a downstream of miR-665 in retinoblastoma [24,25]. In NB, HMGB1 is highly expressed and considered as a tumor-promoting factor [26]. Therefore, we speculated that miR-665 might modulate HMGB1 expression in NB. To confirm their functional interaction, we performed dual-luciferase reporter assay using the construct containing wildtype miR-665 binding sequence of HMGB1 3ʹ UTR (WT-HMGB1) as well as the mutated sequence (MUT-HMGB1). The presence of miR-665 mimic significantly inhibited the luciferase activity of WT-HMGB1, which was abrogated in the MUT-HMGB1 (Figure 4a). We further applied miR-665 mimic and inhibitor to study the role of miR-665 on HMGB1 expression. In both SK-N-BE and SH-SY5Y cells, HMGB1 mRNA and protein levels were significantly decreased in the presence of miR-665 mimic while miR-665 inhibitor increased HMGB1 level (Figure 4b). To confirm that miR-665/HMGB1 is downstream of lncRNA NHEG1, we knocked down lncRNA NHEG1 and found the level of HMGB1 was also decreased (Figure 4c). In addition, miR-665 inhibitor rescued the expression level of HMGB1 after lncRNA NHEG1 knockdown (Figure 4c). These results indicate that lncRNA NHEG1 maintains the expression of HMGB1 by negatively affecting the activity of miR-665.

Furthermore, there was a negative correlation between the expression levels of miR-665 and HMGB1 (Figure 3d), and a positive correlation between the expression levels of lncRNA NHEG1 and HMGB1 in NB tumor sample (Figures 4d, e). Kaplan-Meier curve analysis revealed that high expression of HMGB1 was correlated with a worse overall survival (OS) and progression-free survival (PFS) (Figures 4f, g). Collectively, these data suggest that the expression of lncRNA NHEG1 regulates HMGB1 level by targeting miR-665 in NB tumors.

## LncRNA NHEG1/miR-665/HMGB1 axis is involved in regulating the malignant phenotype of NB cells

**Figure 5.**
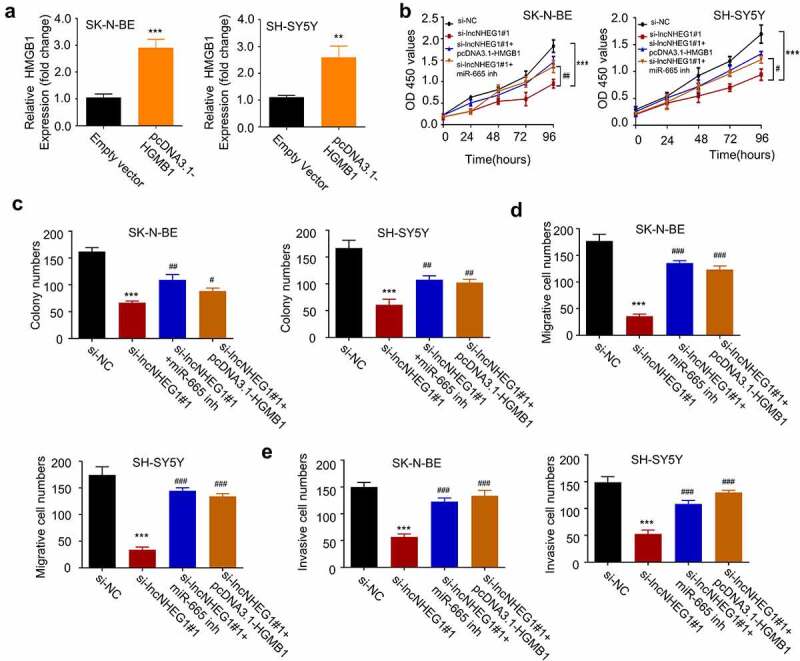
LncRNA NHEG1/miR-665/HMGB1 axis is involved in regulating the malignant phenotype of NB cells A. HMGB1 expression level was determined by qRT-PCR after transfection with pcDNA3.1-HMGB1 in NB cells. B. CCK-8 cell proliferation assay NB cells treated with si-NC, si-lncNHEG1#1, si-lncNHEG1#1+ miR-665 inhibitor, or si-lncNHEG1#1+ pcDNA3.1-HMGB1. C. Colony formation experiment was performed in NB cells with the above treatments. D. Cell migration experiment in NB cells treated with the above treatments. E. Cell invasion experiment in NB cells with the above treatments. All experiments were performed 3 times and data were presented as mean ± SD. **P < 0.01, ***P < 0.001 vs. si-NC group. #*P* < 0.05, ##P < 0.01, ###*P* < 0.001 vs. si-lncNHEG1#1 group

The above results revealed the regulation of HMGB1 by lncRNA NHEG1 and miR-665 in NB. We next sought to validate the functional role of lncRNA NHEG1/miR-665/HMGB1 axis in regulating the malignant phenotype of NB cells. We constructed an expression vector by cloning HMGB1 cDNA into pcDNA3.1 expression plasmid, which could significantly increase HMGB1 expression in both SK-N-BE and SH-SY5Y cells (Figure 5a). We next silenced lncRNA NHEG1 in the presence of pcDNA3.1-HMGB1 or miR-665 inhibitor. CCK-8 proliferation and colony formation assays revealed that the inhibitory effects of lncRNA NHEG1 knockdown on cell proliferation and colony formation were rescued by HMGB1 overexpression or miR-665 inhibitor (Figures 5b, c). Transwell experiments further demonstrated that HMGB1 overexpression or miR-665 inhibitor largely abrogated the inhibition of lncRNA NHEG1 knockdown on cell migration and invasion (Figures 5d, e). Collectively, these data strongly suggest that miR-665/HMGB1 axis mediates the oncogenic effect of lncRNA NHEG1 in NB cells.

## Discussion

We explored the expression level and the functional role of lncRNA NHEG1 in NB. We found that: (1) lncRNA NHEG1 was upregulated in NB and correlated with prognosis of NB patients; (2) downregulation of lncRNA NHEG1 could inhibit the proliferation, migration, and invasion of NB cells; (3) the miR-665/HMGB1 axis, which was also previously reported to regulate retinoblastoma [24,25], may serve as the downstream effectors mediating the role of lncRNA NHEG1 in NB.

The crucial role of lncRNAs in multiple human cancer has been reported by a number of studies [29–33]. As shown by previous studies, lncRNA could directly target tumor suppressors or oncogenic protein to regulate cancer development and progression. For example, lncRNA NHEG1 plays an oncogenic role in NB by physically interacting with and stabilizes DEAD-box helicase 5 (DDX5), which activates β-catenin pathway and drives NB progression [10]. Consistently, we showed that lncRNA NHEG1 may factor the progression of NB since it is upregulated in NB tumor and cell lines. Silencing lncRNA NHEG1 suppresses the aggressive phenotype, indicating that lncRNA NHEG1 is indispensable for supporting the malignant phenotype of NB cells.

A previous study suggests that lncRNA NHEG1 regulates a network of microRNAs in human lung adenocarcinoma [9], implying the involvement of miRNAs in the regulatory network by lncRNA NHEG1. In our study, we showed that miR-665 was downregulated in NB tumors and lncRNA NHEG1 silencing caused its upregulation in NB cells. The upregulation of lncRNA NHEG1 and downregulation of miR-665 have been also reported in neuroblastoma [10,13]. By dual-luciferase reporter assay, we further showed that lncRNA NHEG1 functionally interacted with miR-665. Furthermore, silencing lncRNA NHEG1 led to the upregulation of miR-665, which was accompanied by the inhibition of proliferation, migration, and invasion in NB cells. Therefore, lncRNA NHEG1 serves as an upstream factor negatively affecting the activity of miR-665.

HMGB1 has been implicated in NB and its role seems to be related to autophagy and miRNA regulation [26,34,35]. Recent studies also showed that miR-665 serves as a tumor-suppressor in retinoblastoma by targeting HMGB1^24^[25]. Our data revealed that miR-665 targets HMGB1 mRNA and caused its downregulation in NB cells. This may be related to both mRNA degradation or translational inhibition [13–15]. Dual-luciferase reporter assay further confirmed the functional interaction between miR-665 and HMGB1 mRNA. Importantly, we further showed that miR-665/HMGB1 axis functions downstream of lncRNA NHEG1. Through a series of functional assays, we demonstrated that lncRNA NHEG1 knockdown suppressed cell proliferation, migration, and invasion of NB cells, which was rescued by miR-665 inhibitor and HMGB1 overexpression. Collectively, our data imply that lncRNA NHEG1 serves as a competitive partner to negatively regulate miR-665, which relieves the inhibition on HMGB1 expression and promotes the aggressive phenotype of NB cells. Thus, lncRNA NHEG1/miR-665/HMGB1 axis may regulate NB progression.

However, other downstream effectors of lncRNA NHEG1 may be also implicated in regulating NB cell phenotype, which requires further investigation. Furthermore, the role of lncRNA NHEG1/miR-665/HMGB1 axis in NB progression needs to be validated in animal model.

## Conclusion

In summary, our study demonstrated the upregulation and the functional role of lncRNA NHEG1 in NB tumors and cell line. We further unveiled the involvement of miR-665/HMGB1 axis in the regulation of the malignant phenotype of NB cells, which mediates the downstream effect of lncRNA NHEG1. Collectively, our study suggests that lncRNA NHEG1/miR-665/HMGB1 axis is involved in regulating the aggressiveness NB cells, which may be implicated in the progression of NB.

## Highlights

- LncRNA NHEG1 is upregulated in neuroblastoma (NB) tumor and cell lines.
- hsa-miR-665 (miR-655) serves as a downstream target of lncRNA NHEG1.
- miR-655 negatively regulates the expression of high mobility group box 1 (HMGB1).
- LncRNA NHEG1/miR-665/HMGB1 axis regulates NB cell aggressiveness.
